# Real-Time Footprint Planning and Model Predictive Control Based Method for Stable Biped Walking

**DOI:** 10.1155/2022/4781747

**Published:** 2022-04-01

**Authors:** Song Wang, Songhao Piao, Xiaokun Leng, Zhicheng He, Xuelin Bai, Li Huazhong

**Affiliations:** ^1^School of Computer Science, Harbin Institute of Technology, Harbin, China; ^2^Leju Robotics, Shenzhen, China; ^3^Shenzhen Institute of Information Technology, Shenzhen, China

## Abstract

In order to walk in a physical environment, the biped will encounter various external disturbances, and walking under persistent conditions is still challenging. This paper tries to improve the push recovery performance based on capture point (CP) and model predictive control. The trajectory of zero moment point (ZMP) and center of mass are solved and predicted in a limited time horizon. Online footprint generator is combined with MPC walking pattern generation, which can keep biped stable in the next few steps, and projection of ZMP is used to calculate the next footprint and reach the target CP in an incremental way. Verification of the proposed stable biped walking method is conducted by simulation and experiments.

## 1. Introduction

tBiped robot is one kind of mobile robot and belongs to the field of leg robot. In the world of robotics, biped walking is one of the fields with the most interesting research [1]. Robots need to interact and collide intermittently with the environment to complete specific actions and tasks [2]. Compared with other forms of robots, biped robot has more advantages: the biped with a humanoid structure can adapt to various environments, and it can replace humans in many dangerous jobs and offer services for humans, such as artificial limb and medical rehabilitation [3].

The biped robot model is nonlinear dynamic with multiple degrees of freedom and constraints, and it is important to interpret and predict the long-term behavioral trends of the dynamic system for various control tasks. A simplified biped walking linear inverted pendulum model (LIPM) is the baseline of humanoid walking pattern controller [4, 5], and passive walking and under-actuated robots have certain advantages in energy-saving walking. But passive walking has poor terrain adaptability and can only walk downhill or on flat ground; while under-actuated walking robots require special structural design and rely on complex walking control strategies [6]. Zhang et al. [7] proposed a walking pattern generator for omnidirectional walking on a slope and uneven plane and realized the oblique walk and heading for any direction, but the performance of push recovery under external persistent force is not analyzed. Oliver et al. proposed an improved model named the flexible linear inverted pendulum model (FLIPM) which integrate damped spring and second center of mass to LIP [8], which realized a stable walk on a servo-driven biped robot. However, the performance is only improved in the *z* direction, which is not included by FLIPM and causes a rotation of the root alongside the *x*-axis towards the ankle joint lifting up from the plane. Biped robot has dozens of degrees of freedom and no fixed base, which leads to its dynamic system is very complex. Juang et al. proposed the fully connected recurrent neural network optimized by continuous multiobjective ant colony optimization to form CpG to solve the multiobjective gait generation problem of Nao robot. An open-source multiple DOF servo-driven robot Robotis op [9] is used to test their method performance, but the coupled oscillator model is not able to keep stability under an unpredictable perturbation.

In the process of walking, the biped robot will inevitably be affected by various uncertain factors, such as the uncertainty of mathematical model based on different assumptions [10], the uncertainty of robot system parameters caused by the structural size, material properties, manufacturing and assembly errors, and the random uncertainty of driving torque caused by actuator noise and joint friction, and the external environment disturbance and plane condition uncertainty in the process of robot walking [11]. Pratt et al. [12] introduced the concepts of capture point and capture area by LIPM and flywheel model, and CP is the key metric for push recovery of the biped which shows the target direction to maintaining body stability by keeping center of pressure (CoP) inside the foot support area. But how the capture region adapts with other biped models is not discussed. Majid et al. generate walking patterns for the biped by two stages: computes the best step location and duration and adapts these values using divergent component of motion (DCM) measurement. Englsberger [13] designed CP following and CP step-end controller and has proved the antipush stability of CP control principle, but real-time footprint position adjustment is not included to enhance the robustness of the walking pattern against external perturbations. Since the large number of joints, it is very complex to use CPG model to describe the joint angle for the 3D humanoid robot. Q-learning-based CPG model [14, 15] can keep the body recover from horizon perturbation, but it need different expertise to redesign the controller. In addition, the motion obtained by this gait control method is generally not optimal, and the anti-interference in vertical direction ability is poor.

Humanoid walking pattern generation can be solved by model predictive control (MPC) with efficient constraint handling [16]. The optimization-based method is utilized so as to generate, at each iteration, the optimal gait trajectory of the system satisfying the given constraints. However, it is well known that the action of a bounded persistent disturbance can destabilize a predictive controller which has been designed to be stabilizing for the nominal case [17]. These controllers can be used with a cost function that at the specific problem of generating trajectory by quadratic programming in linear system control, a fast optimization algorithm is proposed. However, several papers like [18–20] used restrictions on model state at the end of the prediction horizon. Since the state of the system is completely dependent on the beginning and the end of the horizon, motions lack the flexibility to perform secondary control objectives. Amos et al. [21] proposed the strategy architecture designed for end-to-end training, the robot learned to combine high-level planning strategy with low-level motion controller to realize autonomous navigation on a curved path, which is less computationally and memory intensive compared to traditional optimization solutions, but the model used in that article is still linear which is hard to extend to complex systems like the biped walking. Scianca et al. [22] introduced an IS-MPC framework for gait generation which extend LIPM with ZMP trajectory input, and recursive feasibility of internal stability is realized for biped dynamics, but the cost of control and response speed of method are not analyzed for position control based humanoid robot [23].

**Figure 1:**
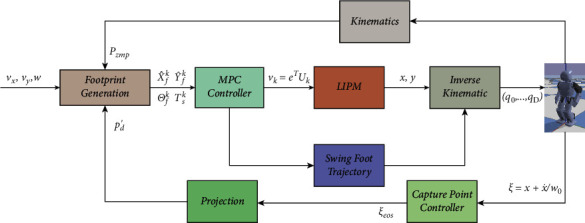
Block scheme of the proposed CP- and MPC-based framework for walking gait.

The gait trajectory planning method based on LIP does not consider the external interference force, so it cannot walk in a contact-rich environment. The gait pattern generator can only calculate the trajectory position at the next time according to the state transition equation but cannot predict the trajectory in the future. This paper proposes footprint planning and MPC method for stable biped walking considering the capture point principle. The framework of the method is summarized in Figure 1. LIPM is used to linearize the dynamic model of biped walking, and MPC is used as a gait pattern generator to generate a smooth trajectory of CoM. Online footstep generator based on capture point feedback is combined with the MPC controller, and the biped can take one or more steps to reach the stable state and realize omnidirectional walking on the horizon plane. And a projection function maps the target ZMP into support polygon which makes inverse kinematics is solvable.

## 2. Materials and Methods

### 2.1. Approximation of Walking Model

Approximation acts effective method to deal with complex systems. Most humanoid robots use the model-based walking planning method to get the gait pattern and abstract the biped dynamics equation from the model by approximating the center of mass (CoM). The classical inverted pendulum model is widely used for humanoid walking pattern generation. Some conditions are mentioned first as follows:

1. Whole-body mass of the robot is concentrated on the CoM.
2. CoM height *h*_*CoM*_ in 3-dimensional space is invariant.
3. Biped legs are massless and contact with the ground through a pivot that can be rotated.

Under the joint action of the supporting moment and the force along the stretching direction of the rod, the dynamic equation of LIPM is established, and the trajectory of the CoM is obtained by solving the dynamic equation. Using the LIPM, let *p* denote the position of the center of pressure (CoP) on the plane, and the horizontal dynamics differential equation of CoM is

(1) $$\begin{array}{c} \ddot{x}=\omega_{0}^{2}\left(x-p\right), \end{array}$$

where $w_{0}=\sqrt{g/h_{CoM}}$ and *g* is gravity force, and *h*_*CoM*_ is the constant height of CoM along *z*-axis. *x* denotes the position of CoM along *x*-axis, and $\ddot{x}$ denotes body acceleration. This approximation (1) decouples the sagittal and coronal motions of the biped robot, so we will focus on the *x*-axis motion throughout this paper, and *y* direction motion is totally identical.

Considering the discretization at a minimum interval time *T*, CoM and CoP are discretized. Let input control signal $u\left(t\right)=\dddot{x}$ which is the jerk of CoM position in state-space equation, then the system state transition at time *t*=*kT*, *k*=1,2, ... with notation

(2) $$\begin{array}{c} {\hat{x}}_{k}=\left[\begin{array}{c} x\left(kT\right)\\ \dot{x}\left(kT\right)\\ \ddot{x}\left(kT\right) \end{array}\right],u_{k}=\dot{x}\left(kT\right),p_{k}=p\left(kT\right). \end{array}$$

From (2), we can get the ZMP position

(3) $$\begin{array}{c} p_{k}=\left[\begin{array}{ccc} 1 & 0 & 1 \end{array}/w_{0}^{2}\right]{\hat{x}}_{k}. \end{array}$$

For the system state ${\hat{x}}_{k}$, the discretized system of LIPM is

(4) $$\begin{array}{c} {\hat{x}}_{k+1}=\left[\begin{array}{ccc} 1 & T & T^{2}/2\\ 0 & 1 & T\\ 0 & 0 & 1 \end{array}\right]{\hat{x}}_{k}+\left[\begin{array}{c} T^{3}/6\\ T^{2}/2\\ T \end{array}\right]u_{k}\\ =A{\hat{x}}_{k}+Bu_{k}. \end{array}$$

In the process of bipedal walking, the position of ZMP *p*_*k*_ always falls into the support polygon of the foot, so the range of ZMP can be limited within

(5) $$\begin{array}{c} p_{k}^{\text{min}}<p_{k}<p_{k}^{\text{max}}. \end{array}$$

The range of ZMP [*p*_*k*_^min^, *p*_*k*_^max^] depends on foot contact condition with the horizontal plane at time *kT*. According to the state transition (2), the next state at time *k*+1 is decided by the current state *x*_*k*_ and input control *u*_*k*_. The core idea of MPC makes the actual ZMP *p*_*k*_ most likely close to reference ZMP trajectory *p*_*k*_^*ref*^ at the minimum control cost at time *kT*. Then, the optimal control sequence *u*_*k*_, *u*_*k*+1,..._ can be get by solving the quadratic problem (QP).

(6) $$\begin{array}{c} \underset{u_{k},u_{k+1},\ldots }{\min}\sum_{i=k}^{\infty }\frac{1}{2}\alpha {\left(p_{i+1}-p_{i+1}^{ref}\right)}^{2}+\frac{1}{2}\beta u_{i}^{2}, \end{array}$$

where *α*/*β*(*α* > 0, *β* > 0) are factors to balance the system response ratio and control cost. If *α*/*β* rises, the tacking speed of system and control costs increase. And if *α*/*β* is lower, the tracking speed of the system and control costs decrease.

### 2.2. Capture Point Controller

**Figure 2:**
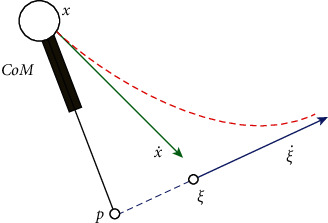
Top view of the LIPM with point foot for a given initial state.

For push resistance of the bipedal, instantaneous capture point (ICP) was utilized. During the motion of a LIP model, there exists a metric point on a walking plane, where placing the center of the landing foot can stabilize the motion of the model. This point can be calculated as

(7) $$\begin{array}{c} \xi =x+\frac{\dot{x}}{w_{0}}, \end{array}$$

where *ξ* is the *x* or *y* component of ICP position on plane. Due to the limitation of robot dynamics, when the capture point is not captured, the CP will be away from CoP in a straight line until it falls down that is noted by Figure 2.

The features of the capture point can be described as follows:

1. Due to the regular dynamics of the robot, if the point is not captured, with time, the ICP moves in a straight line joining the CoP and the CP in a direction away from the foot, as shown in Figure 2.
2. Zero-step capturability: If the ICP falls inside the support polygon, then the robot can balance itself with the application of a good control like MPC; however, if the ICP falls outside the support polygon, the robot will have to take at least one step to protect itself from falling down.
3. One-step capturability: The model can come to stability within a step if the ICP falls within the reachable range. The reachable region is bounded by the maximum step length.

According to equation (1) and equation (7), the dynamic relationship between CP and ZMP is

(8) $$\begin{array}{c} {\dot{\xi }}_{x}=\omega_{0}\left(\xi_{x}-p_{x}\right). \end{array}$$

Since the pole of transition function is *ω*_0_, equation (7) is an unstable system without external additional input. The idea of CP control is to generate the ZMP trajectory so that the current CP reaches the target CP within a given time interval, and then, the CoM follows the planning CP curve. The solution of equation (7) in the time domain is

(9) $$\begin{array}{c} \xi_{x}\left(t\right)=e^{\omega_{0}t}\left(\xi_{x,0}-p_{x}\right)+p_{x}, \end{array}$$

where *ξ*_*x*,0_ is the initial position of CP. It can be seen that when ZMP *p*_*x*_ is constant, CP will change exponentially. Let *ξ*_*x*,*d*_, *ξ*_*x*_ represent the target CP and current CP, respectively, and time from *ξ*_*x*,*d*_ to *ξ*_*x*_ is *dT*, and then, (9) can be discretized as

(10) $$\begin{array}{c} p_{x}=\frac{\xi_{x,d}-e^{w_{0}dT}\xi_{x}}{1-e^{w_{0}dT}}=\frac{1}{1-e^{w_{0}dT}}\xi_{x,d}-\frac{e^{w_{0}dT}}{1-e^{w_{0}dT}}\xi_{x}. \end{array}$$

The goal of end-of-step CP controller: ZMP trajectory is generated by *ξ*_*x*,*d*_ and variable time span *dT*. Let *ξ*_*x*,*eos*_ represent the end of step at the ending of each foothold, and *ξ*_*x*,*d*_=*ξ*_*x*,*eos*_. Suppose the center of the support foot is approximately regarded as the ZMP point *p*_*i*_ at step *i*, and the initial CP is

(11) $$\begin{array}{c} \xi_{init,i}=p_{i}+\left(\xi_{eos,i}-p_{i}\right)/e^{w_{0}t_{step}}, \end{array}$$

where *t*_*step*_ is the duration of each step. Iterative relation of CP state is *ξ*_*eos*,*i*−1_=*ξ*_*init*,*i*_. When the robot is subjected to external thrust, CP changes suddenly, and the biped needs to replan the landing footprint so that the CoM is adjusted to *ξ*_*eos*_. According to the iterative relationship (11), the CP position in each support leg cycle can be calculated. Then, the system reaches a stable state following the new reference ZMP sequence generated by the CP controller.

ZMP reference trajectory is a continuous curve and constrained by robot dynamics of support polygon. When the system is disturbed by a large external force, CP varies fast out of feet support polygon on the plane. If *p*_*x*_ is calculated by (11), ${\hat{p}}_{d}$ might locate out of support polygon. And the incremental progressive method can be adopted for ZMP, and CoM is controlled to move along the target CP direction within the current support polygon. Formula (9) is differentiated as follows:

(12) $$\begin{array}{c} d\xi_{eos}=\frac{\partial \xi_{eos}}{\partial p_{d}}=\left(1-e^{\omega_{0}dT}\right)dp_{d},\\ \Delta \xi_{eos}=\int d\xi_{\mathrm{eos}}dt. \end{array}$$

**Figure 3:**
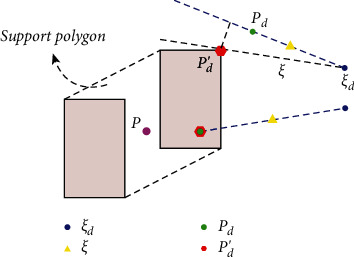
Projection of *p*_*d*_ in support polygon.

A method to implement the incremental integration (12) is the ZMP projection as shown in Figure 3. If the projection is not applied, *p*_*d*_ from (11) could beyond the support polygon, and the CoM trajectory generated by *p*_*d*_ will cause falling on the ground.

As Figure 3 shows, we found a new projection ZMP *p*_*d*_′ in support polygon to make CoM follow the target direction of CP. The projection rules can be summarized as follows:

- If *p*_*d*_ is located in support polygon, the target ZMP *p*_*d*_ is in the reachable region of the biped, then projection ZMP *p*_*d*_′=*p*_*d*_.
- If *p*_*d*_ is located outside the support polygon, *p*_*d*_′ is the closest point on the inner boundary of the support polygon to (*ξ*_*d*_ − *ξ*).

In practical application, the number of reverse iterative footprint calculation *F* is limited. Based on the current foothold *p*_*i*_ and initial CP *ξ*_*init*,*i*_, the position of ZMP can be solved on a limited horizon *F*. CoM mapping is used to make the CoM approach the target position. But as 3 shows, there is a discontinuity between current *p* and *p*_*d*_′=*p*_*d*_ which leads to discontinuous input to the walking pattern generator. To compensate for this problem, the MPC controller is introduced in the next section to track the CP trajectory on the one hand and export smooth CoM trajectory, so that the biped will not lose stability due to the sudden change of dynamic parameters in the process of walking.

### 2.3. Footprint Generator with Variable Velocity

The footprint generator module and MPC controller module are implemented synchronously. The input of the footprint generator is reference velocity *v*_*x*_, *v*_*y*_ and angular velocity *w* around the *z*-axis. The output of this generator is the next footprint position, direction, and time. With the change of input reference velocity, the cycle and step length of biped walking will be affected, and the footprint generator calculates the next foothold position by solving the quadratic optimization problem. The time and orientation of the foothold of the output of the footprint generator will be used as the reference input of the MPC controller in the next step.

As shown in Figure 1, the input of the footprint generator is *v*_*x*_, *v*_*y*_, *w* from time *t*_*k*_ to *t*_*k*+*P*_=*t*_*k*_+*T*_*p*_, where *T*_*p*_ is the duration of the footstep. The output of the footprint generator is the sequence $\left({\hat{X}}_{f}^{k},{\hat{Y}}_{f}^{k},\Theta_{f}^{k}\right)$ on time sequence *𝒯*_*s*_^*k*^, and

(13) $$\begin{array}{c} {\hat{X}}_{f}^{k}={\left(x_{f}^{1}...x_{f}^{F}\right)}^{T},\\ {\hat{Y}}_{f}^{k}={\left(y_{f}^{1}...y_{f}^{F}\right)}^{T},\\ \Theta_{f}^{k}={\left(\theta_{f}^{1}...\theta_{f}^{F}\right)}^{T},\\ T_{s}^{k}=\left\{T_{s}^{1},...,T_{s}^{F}\right\}, \end{array}$$

where *F* is the number of next footprints, and (*x*_*f*_^*j*^, *y*_*f*_^*j*^, *θ*_*f*_^*j*^) is the position and orientation of *j*-th footprint. *T*_*s*_^*j*^ is the duration from *j* − 1-th to *j*-th footprint. Since the input velocity is variable, the prediction horizon of time *T*_*s*_ is constant, and ∑_j=1_^F^*T*_*s*_^*j*^=*T*_*p*_, and then, distribution of (*x*_*f*_^*j*^, *y*_*f*_^*j*^, *θ*_*f*_^*j*^) is nonequidistant during *T*_*p*_.

Assume that the biped is within the j-th support phase switching duration *T*_*s*_^*j*^, $\bar{v}$ is average velocity, ${\bar{T}}_{s}$ is step period duration, and ${\bar{L}}_{s}$ is stride length, so $\bar{v}={\bar{L}}_{s}/{\bar{T}}_{s}$. Walking velocity is limited by the biped dynamics such as CoM height, degree of freedom, and leg length. $\bar{v}$ is determined by both *T*_*s*_^*j*^ and ${\bar{L}}_{s}$. Δ*v* is velocity small variation in Δ*t*, and then,

(14) $$\begin{array}{c} v=\bar{v}+\Delta v=\frac{{\bar{L}}_{s}+\Delta L_{s}}{{\bar{T}}_{s}-\Delta T_{s}}, \end{array}$$

where Δ*L*_*s*_=*γ*Δ*T*_*s*_, and (14) can be expressed as

(15) $$\begin{array}{c} T_{s}={\bar{T}}_{s}\frac{\gamma +\bar{v}}{\gamma +v}. \end{array}$$

**Figure 4:**
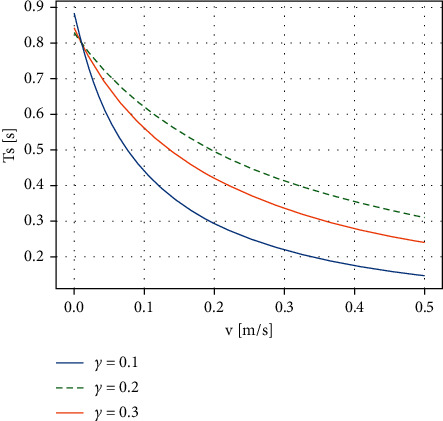
Rules for step duration *T*_*s*_ and velocity $\bar{v}$, and comparison of different *γ*.

Let $\bar{v}=0.01m/s$, ${\bar{T}}_{s}=0.8s$, and the relationship between velocity and duration can be shown in Figure 4.

During a small time interval *δt*, velocity can be approximately regarded as a linear variation. Therefore, the orientation angle of the robot can be approximately constant, and angular velocity is ignored in (14).

Considering time sequence *t*_2_^1^, *t*_2_^2^, ..., *t*_2_^*F*^ iterative relation, the time iterative equation within [*t*_*k*_, *t*_*k*_+*T*_*p*_] is

(16) $$\begin{array}{c} t_{s}^{j}=t_{s}^{j-1}+{\bar{T}}_{s}\frac{\gamma +\bar{v}}{\gamma +v\left(t_{s}^{j-1}\right)}, \end{array}$$

where *t*_*s*_^0^ is the end time of the last support leg duration, and the iteration is over when *j* > *k*+*P*. Then, a time sequence *𝒯*_*s*_^*k*^={*T*_*s*_^1^, ..., *T*_*s*_^*F*^} is outputted with *T*_*s*_^*j*^=*t*_*s*_^*j*+1^ − *t*_*s*_^*j*^.

According to the omnidirectional motion model of a mobile robot, the bipedal moves to the target point at any angle and direction in the horizontal plane with *v*_*x*_, *v*_*y*_, *w*.

(17) $$\begin{array}{c} \left(\begin{array}{c} \dot{x}\\ \dot{y}\\ \dot{\theta } \end{array}\right)=\left(\begin{array}{ccc} \text{cos}\theta & -\text{sin}\theta & 0\\ \text{sin}\theta & \text{cos}\theta & 0\\ 0 & 0 & 1 \end{array}\right)\left(\begin{array}{c} v_{x}\\ v_{y}\\ \omega \end{array}\right). \end{array}$$

The idea of the footprint generator is to allocate the future foothold under the condition of considering the constraints of kinematics and dynamics parameters of the biped in time *𝒯*_*s*_^*k*^={*T*_*s*_^1^, ..., *T*_*s*_^*F*^}. When the body root is subjected to push forces, according to (7), $\dot{x}$ will change suddenly, which will affect the planned trajectory of *x*. The velocity and acceleration change suddenly, which affect the planning trajectory of CoM. The position of the foothold need be adjusted to make the CoM as close as possible near new CP so that the CoM can keep a gradually stable state.

**Figure 5:**
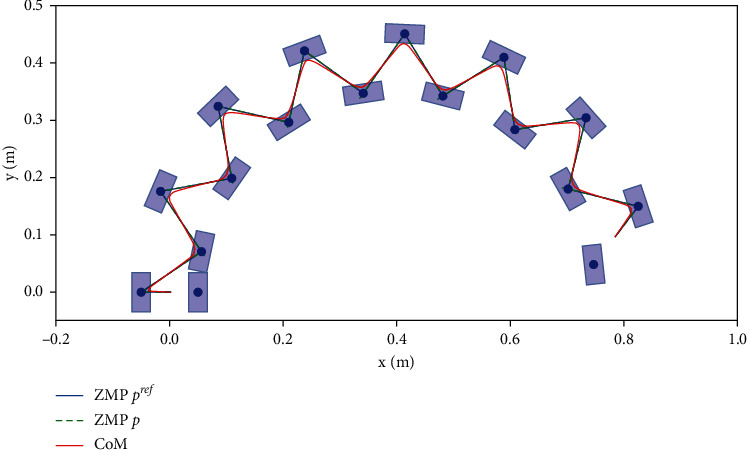
Candidate footsteps generated by the footprint generator in half circle demonstrate the omnidirectional feature.

The footprint planning can be transformed into two QP optimization problems as follows:

(18) $$\begin{array}{c} \left\{\begin{array}{c} \underset{\Theta_{f}^{k}}{\min}\sum_{j=1}^{F}{\left(\theta_{f}^{j}-\theta_{f}^{j-1}-\int_{t_{s}^{j-1}}^{t_{s}^{j}}\omega \left(\tau \right)d\tau \right)}^{2}\\ st.\left|\theta_{f}^{j}-\theta_{f}^{j-1}\right|\leq \theta_{\max} \end{array}\right., \end{array}$$

where *θ*_max_ is the maximum steering angle allowed for two consecutive footprints according to the physics dynamics of the biped. And the second QP problem is

(19) $$\begin{array}{c} \underset{{\hat{x}}_{f}^{k},{\hat{r}}_{f}^{k}}{\min}\sum_{j=1}^{F}{\left({\hat{x}}_{f}^{j}-{\hat{x}}_{f}^{j-1}-\Delta x^{j}\right)}^{2}+{\left({\hat{y}}_{f}^{j}-{\hat{y}}_{f}^{j-1}-\Delta y^{j}\right)}^{2}. \end{array}$$

where the supporting foot position is $\left({\hat{x}}_{f}^{0},{\hat{y}}_{f}^{0}\right)$ at the starting time *t*_*k*_. The output of the footprint generator (X_*f*_^*k*^, Y_*f*_^*k*^, Θ_*f*_^*k*^) is the input of the MPC controller, and the final ZMP and CoM trajectory is calculated by MPC.  Figure 5 shows an example of footprint generation where the orientation of the robot coincides with the tangent of the motion path. The swing foot trajectory planning is simple, and in order to avoid sudden changes in the velocity of end point, we use Bezier curves to generate swing foot trajectory and then solve the joints rotation by inverse kinematics.

### 2.4. MPC Controller

Predictive control approximates the robot to a three-dimensional linear inverted pendulum, introduces the state space method, optimizes the performance index by the linear quadratic regulator, and limits the movement of the CoM in the horizontal plane with constant height. The state feedback gain and predictive gain of the predictive controller are generated by solving discrete algebraic Riccati equations. Due to the consideration of state feedback, error feedback, and future target information, a stable walking mode can be generated with less computation.

The system only performs prediction of MPC controller at *kT*, then measures the actual state $x,\dot{x}$ of the system as feedback by LIPM, and calculates the optimal control by solving QP. *N* represents the prediction horizon of the MPC controller, and equation (6) can be rewritten within [*kT*, (*k*+*N*)*T*].

(20) $$\begin{array}{c} \underset{u_{k},\ldots ,l_{k+N}}{\min}\sum_{i=k}^{k+N-1}\frac{1}{2}\alpha {\left(p_{i+1}-p_{i+1}^{ref}\right)}^{2}+\frac{1}{2}\beta u_{i}^{2}. \end{array}$$

The above equation (20) is solved by solving complex algebraic Riccati equation in [5], but this method has low computational efficiency. Within MPC prediction horizon, equation (4) can be transformed to matrix operation. The state transition equation is rewritten with length *N* as

(21) $$\begin{array}{c} \begin{array}{c} \left[\begin{array}{c} p_{k+1}\\ \vdots \\ p_{k+N} \end{array}\right]=\left[\begin{array}{ccc} 1 & T & T^{2}/2-1/w_{0}^{2}\\ \vdots & \vdots & \vdots \\ 1 & NT & N^{2}T^{2}/2-1/w_{0}^{2} \end{array}\right]{\hat{x}}_{k}\\ +\left[\begin{array}{ccc} T^{3}/6-T/w_{0}^{2} & 0 & 0\\ \vdots & \ddots & 0\\ \left(1+3N+3N^{2}\right)T^{3}/6-T/w_{0}^{2} & \cdots & T^{3}/6-T/w_{0}^{2} \end{array}\right]\\ \times \left[\begin{array}{c} u_{k}\\ \vdots \\ u_{k+N-1} \end{array}\right], \end{array} \end{array}$$

And (21) can be abbreviated as

(22) $$\begin{array}{c} P_{k+1}=Q_{x}{\hat{x}}_{k}+R_{u}U_{k}. \end{array}$$

According to (20), the QP equation can be represented as

(23) $$\begin{array}{c} \underset{x_{k}}{\min}\frac{1}{2}\alpha {\left(P_{k+1}-P_{k+1}^{ref}\right)}^{2}+\frac{1}{2}\beta U_{k}^{2}, \end{array}$$

And a standard QP form can be generated by substituting (22) into (23)

(24) $$\begin{array}{c} \underset{U_{k}}{\min}\left[\frac{1}{2}\left(\alpha R_{u}^{2}+\beta \right)U_{k}^{2}+\alpha \left(Q_{x}{\hat{x}}_{k}R_{u}-P_{k+1}^{ref}R_{u}\right)U_{k}\right]. \end{array}$$

According to the definition of MPC, *u*_*k*_ at the current moment is the first prediction of MPC prediction trajectory; that is,

(25) $$\begin{array}{c} u_{k}=e^{T}U_{k}, \end{array}$$

where *e*^*T*^=[1,0, ..., 0]^*T*^, and according to the state formula of LIPM (4), the trajectory of *x* and output position of ZMP at the next moment are calculated as

(26) $$\begin{array}{c} {\hat{x}}_{k+1}=A{\hat{x}}_{k}+Bu_{k}=A{\hat{x}}_{k}+Be^{T}U_{k}. \end{array}$$

Within the predictive horizon *NT*, a balance between response speed and control cost of system can be found by adjusting *α*/*β*(*α* > 0, *β* > 0), and in this article, *α*/*β*=10^−5^. When the system and environment models are well known, *NT* can approach infinity. But LIPM model itself is a linear approximation to the walking dynamics of biped robot with systematic model errors, and environment cannot be accurately modeled. So prediction horizon could not be infinite. In fact, when *N* increases, the gain of the system is close to 0, which means that the influence of the predicted future value on the stability of the current system is reduced, but it will significantly decrease the computational efficiency of QP calculation. Therefore, this paper sets the MPC control horizon to *NT*=1*s*.

## 3. Results

**Figure 6:**
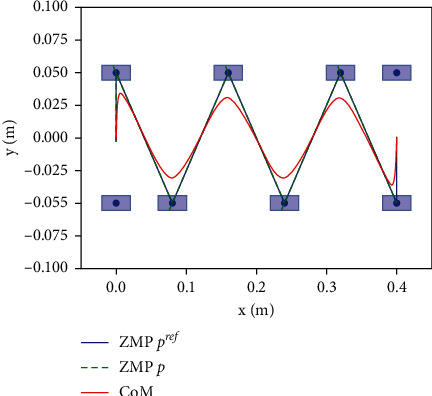
The CoM and ZMP trajectory when walking in a straight line *N*_*step*_=6.

The performance of the proposed dynamic balance control framework in Figure 1 is proved by simulation experiment, we present simulation results using the LIPM and MPC simulation in bullet environment, the input velocity is constant, the single leg support phase time is constant *t*_*sup*_=0.5*s*, supporting leg switching time *t*_*sw*_=0.33*s*, MPC horizon *N*=30, and the minimum control frequency is 100 Hz. The external push force *f*_*ext*_=0, and the robot is not subject to external interference, so that walks along a straight line with *N*_*step*_=6, and according to the foothold generator, the robot's foothold position is shown in Figure 6.

**Figure 7:**
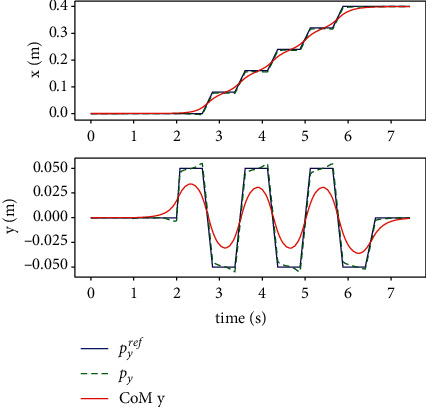
When walking in a straight line, the CoM in the *X* direction (up) and *Y* direction (down) and ZMP trajectory.

Since the LIPM is decoupled from each other in *X* and Y directions, body CoM and ZMP are solved separately by the MPC controller, as is shown in Figure 7. Although there is slight noise jitter in the output ZMP trajectory, the CoM maintains smooth transition, and there is no big speed mutation when the support legs are exchanged. Therefore, the MPC method has good tracking performance.

For the omnidirectional walking, Figure 5 shows velocity direction of walking can be changed in real time by the footprint generator, and *θ*_max_=*π*/4. The robot walks around the center of the circle along with a radius of 2m. The system receives high-level control input *v*_*x*_, *v*_*y*_, *w*, and the optimization problems (18)and (19) is solved at each step. By solving the inverse kinematics, the next step foothold rotation is realized by the hip joint rotation; thus, the omnidirectional walking on the horizon plane of the biped is implemented.

**Figure 8:**
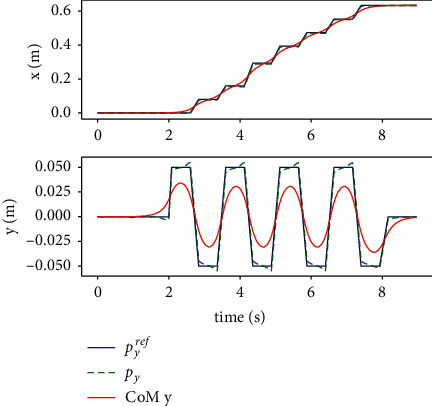
Simulation of tracking the trajectory of time with disturbance force in the sagittal direction.

**Figure 9:**
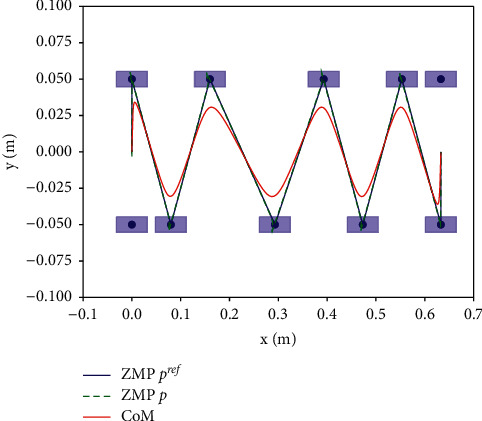
Simulation of tracking the trajectory in horizon plane with disturbance force in the sagittal direction.

**Figure 10:**
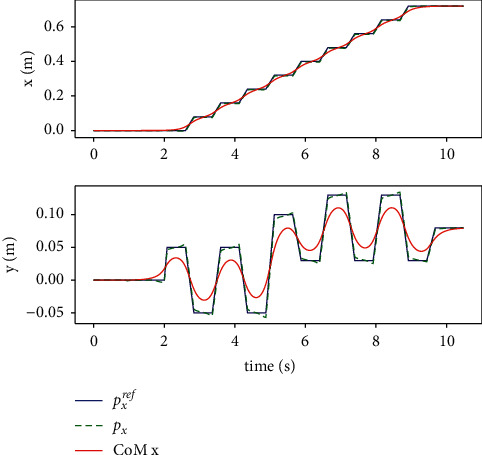
Simulation of tracking the trajectory of time with disturbance force in the coronal direction.

**Figure 11:**
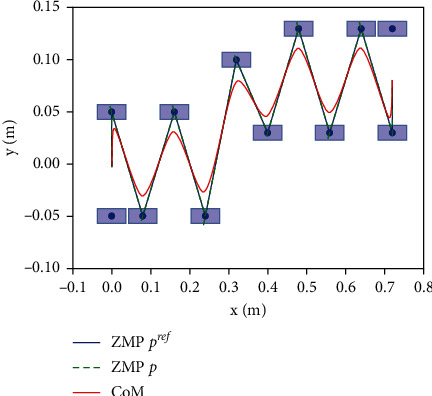
Simulation of tracking the trajectory in horizon plane with disturbance force in the coronal direction.

The performance of the proposed method is measured in case of external disturbance in x-y plane, as shown in Figures 8–11. An instantaneous external thrust *f*_*ext*_=10*N* is added to the CoM of the robot between 4*s* and 5*s*, that duration is 0.01 s. It can be seen that the system can adjust the foothold position in 1 step in both directions and be stabilized.

Since the interference of external thrust, the biped will deviate from the predetermined trajectory, but the velocity and direction of biped walking can be changed in real time, and the system has the ability to adjust the input parameters to make the biped walking towards the target position.

## 4. Conclusions

At the first, a simplified LIPM is used to linearize the dynamic model of biped walking, MPC is used as a gait pattern generator to keep dynamics and kinematics feasibility, and CoM and ZMP trajectory of the biped is solved and predicted in a limited horizon. Since the bipedal system is an unstable system, it is easy to be disturbed by external thrust, resulting in system instability and falling down. Therefore, this paper designs a bipedal balance control method considering capture point with MPC controller, which realizes the footprints planning with variable speed, so that the robot can walk in omnidirectional directions on the plane. At the same time, the capture point parameters of the system are introduced as feedback. When the system is disturbed by external thrust, the acquisition point will change suddenly. By controlling the ZMP point trajectory, the centroid can track the direction of the capture point, and the system will walk to keep it stable. However, CP may fall outside the support polygon. We use a projection method to make the next landing footprint always fall in the range of the biped reachable area and reach the target CP in an incremental manner. For solving the problem of ZMP input point discontinuity caused by projection, the system uses the MPC method to predict the smooth trajectory curve of centroid in the future according to the new foothold position. The position of joint of the full body is calculated by inverse kinematics and drives the biped realized stable walking.

We only focus on the dynamic walking of bipedal on the horizontal plane. It is necessary to analyze the dynamic walking of bipedal on the nonstationary ground and extend the planning of foothold in a two-dimensional plane to three-dimensional space. On the other hand, the gait pattern generator is mainly a linear inverted pendulum model, which is an approximation of bipedal walking and can not plan complex action behavior. And there will be some improvements in the system approximation model, and a more accurate dynamic and environmental model will be used to predict and analyze the robot's future actions and behaviors.

## Data Availability

The data used to support the findings of this study are available from the corresponding author upon request.

## References

[1] Juang C.-F., Yeh Y.-T. (2017). Multiobjective evolution of biped robot gaits using advanced continuous ant-colony optimized recurrent neural networks. *IEEE Transactions on Cybernetics*.

[2] Wieber P.-B., Tedrake R., Kuindersma S. (2016). Modeling and control of legged robots. *Springer Handbook of Robotics*.

[3] Yang X., She H., Lu H., Fukuda T., Shen Y. (2017). State of the art: bipedal robots for lower limb rehabilitation. *Applied Sciences*.

[4] Kajita S., Fumio K., Kenji K., Kazuhito Y., Hirohisa H. The 3D linear inverted pendulum mode: a simple modeling for a biped walking pattern generation.

[5] Park J., Youngil Y. General ZMP preview control for bipedal walking.

[6] Luo J.-w., Fu Y.-l., Wang S.-g. (2017). 3D stable biped walking control and implementation on real robot. *Advanced Robotics*.

[7] Yu Z., Chen X., Huang Q. (2016). Gait planning of omnidirectional walk on inclined ground for biped robots. *IEEE Transactions on Systems, Man, and Cybernetics: Systems*.

[8] Lanari L., Oliver U., Seth H., Ingmar S. (2016). Boundedness approach to gait planning for the flexible linear inverted pendulum model. *In Robot World Cup*.

[9] Li X., Yangmin L., Xinzhe C. Kinematic analysis and gait planning for a Darwin-OP Humanoid Robot.

[10] Joe H.-M., Oh J.-H. (2018). Balance recovery through model predictive control based on capture point dynamics for biped walking robot. *Robotics and Autonomous Systems*.

[11] Gritli H., Belghith S. (2016). Identification, stability and stabilization of limit cycles in a compass-gait biped model via a hybrid poincaré map. *Advances and Applications in Nonlinear Control Systems*.

[12] Pratt J., John C., Sergey D., Ambarish G. Capture point: a step toward humanoid push recovery.

[13] Englsberger J., Christian O., Máximo A. R., Alin A.-S., Gerhard H. Bipedal walking control based on capture point dynamics.

[14] Tran D., Fred H., John N. (2018). A humanoid robot learns to recover perturbation during swinging motion. *IEEE Transactions on Systems, Man, and Cybernetics: Systems*.

[15] Missura M., Sven B. Gradient-driven online learning of bipedal push recovery.

[16] Parsa M., Mohammad F. Robust nonlinear model predictive trajectory free control of biped robots based on nonlinear disturbance observer.

[17] Smaldone F. M., Nicola S., Valerio M., Leonardo L., Giuseppe O. Gait generation using intrinsically stable MPC in the presence of persistent disturbances.

[18] Diedam H., Dimitar D., Pierre-Brice W., Katja M., Moritz D. Online walking gait generation with adaptive foot positioning through linear model predictive control.

[19] Herdt A., Nicolas P., Pierre-Brice W. Walking without thinking about it.

[20] Herdt A., Holger D., Pierre-Brice W., Dimitar D., Katja M., Moritz D. (2010). Online walking motion generation with automatic footstep placement. *Advanced Robotics*.

[21] Amos B., Ivan D. J. R., Jacob S., Byron B., Zico Kolter J. (2018). Differentiable mpc for end-to-end planning and control. http://arXiv.org/abs/1810.13400.

[22] Scianca N., Simone D. De., Lanari L., Oriolo G. (2020). MPC for humanoid gait generation: stability and feasibility. *IEEE Transactions on Robotics*.

[23] Juang C.-F., Yeh Y.-T. (2018). Multiobjective evolution of biped robot gaits using advanced continuous ant-colony optimized recurrent neural networks. *IEEE Transactions on Cybernetics*.

